# The complete chloroplast genome sequence of *Sphagneticola trilobata* (Asteraceae)

**DOI:** 10.1080/23802359.2018.1483767

**Published:** 2018-07-03

**Authors:** Yubing Zhou, Wei Lun Ng, Renchao Zhou, Wei Wu

**Affiliations:** State Key Laboratory of Biocontrol, Guangdong Provincial Key Laboratory of Plant Resources, School of Life Sciences, Sun Yat-Sen University, Guangzhou, China

**Keywords:** *Sphagneticola*, Asteraceae, biological invasion, complete chloroplast genome, automated assembly

## Abstract

*Sphagneticola trilobata* is one of the world's worst invasive alien species. The paucity of genetic information for this species has made it difficult for studies on the underlying genetic mechanism of its invasiveness. Herein, we report the *de novo* chloroplast genome assembly of *S. trilobata* using Illumina whole-genome sequencing data. The chloroplast genome was 151,939 bp in length, with a large single copy (LSC) region of 83,405 bp, a small single copy (SSC) region of 18,448 bp, separated by two inverted repeat (IR) regions of 25,044 bp each. A total of 134 genes were annotated for the chloroplast genome assembly, including 86 protein-encoding genes. Phylogenetic analysis suggested close relationship between *S. trilobata* and *Eclipta prostrata.*

*Sphagneticola trilobata* (L.) Pruski (synonym: *Wedelia trilobata*) is a creeping perennial herb native to South America. It is now commonly found in many tropical or subtropical regions around the world and is listed by IUCN as one of the world’s 100 worst invasive alien species (Lowe et al. 2000). With rampant growth, *S. trilobata* often compete the native species for nutrients, light, and water, sometimes even leading to the decay of their native congeneric species. For instance, natural hybridization between *S. trilobata* and the other indigenous species, *S. calendulacea*, has almost wiped out the latter in South China in the past few decades (Wu et al. 2013). As a step towards understanding the genetic mechanism of invasiveness, we report the complete chloroplast genome sequence of *S. trilobata*, providing some genetic resources for future studies.

The individual used for sequencing was sampled from the campus of Sun Yat-sen University, Guangdong, China. The voucher specimen was deposited at the Sun Yat-sen University Herbarium (SYS) with accession number STR-2017-SYS1. Total DNA was extracted from fresh leaves and used for library construction. A 500-bp insertion library was constructed and paired-end sequenced (125 bp) on a Hiseq2500 platform. Using the *rbc*L gene sequence (KP996856.1) from *S. trilobata* as seed, about 4 GB raw reads were assembled into an intact chloroplast genome using the organelle assembler NOVOPlasty (Dierckxsens et al. 2017). The genome assembly was annotated with DOGMA (Wyman et al. 2004) and manually corrected.

The complete chloroplast genome sequence of *S. trilobata* (GenBank accession KY940274) was 151,939 bp in length, with a large single-copy (LSC) region of 83,405 bp, a small single-copy (SSC) region of 18,448 bp, interleaved by two inverted repeat (IR) regions of 25,044 bp each. The genome comprised of 134 genes, including 86 protein-coding genes, 40 tRNA genes, and 8 rRNA genes. The overall GC content was 37.47%. For phylogenetic tree construction, the chloroplast genome sequences of 11 other representative species in Asteraceae, as well as *Adenophora stricta* in the family Campanulaceae as outgroup, were downloaded from NCBI GenBank. Alignment was performed on the 13 chloroplast genome sequences using MAFFT v7.307 (Katoh and Standley 2013), and a maximum likelihood tree was constructed using RAxML (Stamatakis 2014) with the GTR + G model. *S. trilobata* was shown to be sister to *Eclipta prostrata* in the Heliantheae tribe (Figure 1).

**Figure 1.**
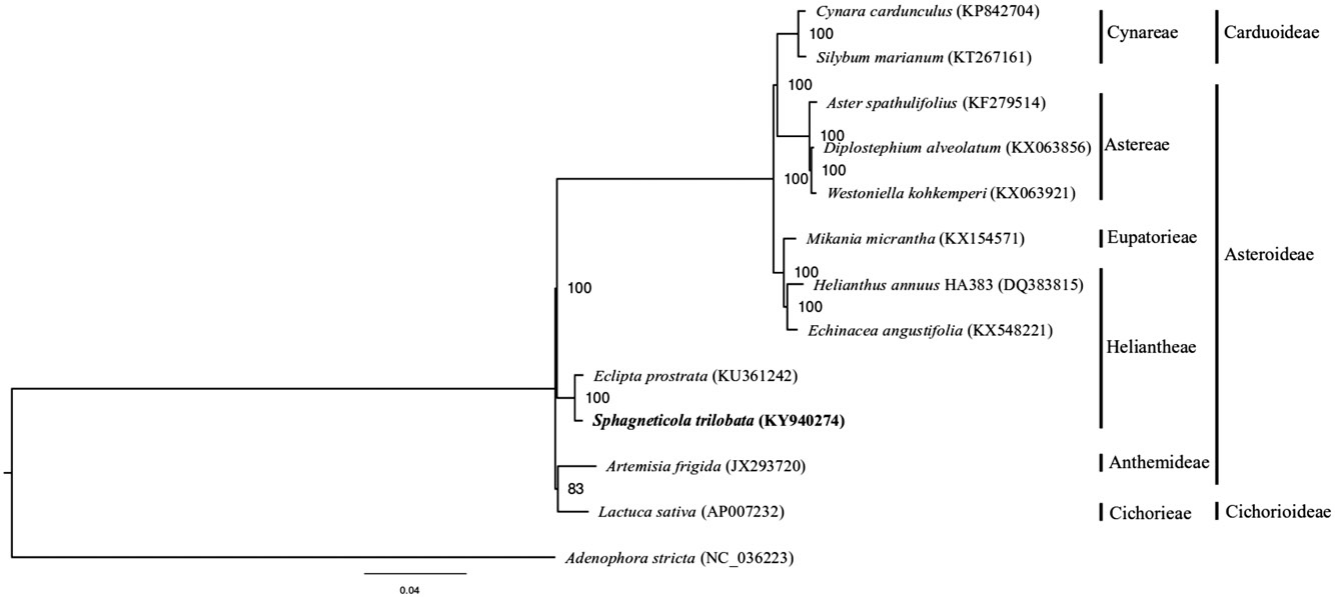
Maximum likelihood tree of *S. trilobata* and other Asteraceae species based on whole chloroplast genome sequence, with *A. stricta* as outgroup. Bootstrap support values (based on 1000 replicates) are shown next to the nodes. Scale in substitutions per site.
